# Statistical Estimation of the Protein-Ligand Binding Free Energy Based On Direct Protein-Ligand Interaction Obtained by Molecular Dynamics Simulation

**DOI:** 10.3390/ph5101064

**Published:** 2012-09-28

**Authors:** Yoshifumi Fukunishi, Haruki Nakamura

**Affiliations:** 1 Biological Information Research Center (BIRC), National Institute of Advanced Industrial Science and Technology (AIST), 2-3-26, Aomi, Koto-ku, Tokyo 135-0064, Japan; 2 Institute for Protein Research, Osaka University, 3-2 Yamadaoka, Suita, Osaka 565-0871, Japan; Email: harukin@protein.osaka-u.ac.jp

**Keywords:** protein-ligand docking, molecular dynamics simulation, protein-ligand binding free energy

## Abstract

We have developed a method for estimating protein-ligand binding free energy (ΔG) based on the direct protein-ligand interaction obtained by a molecular dynamics simulation. Using this method, we estimated the ΔG value statistically by the average values of the van der Waals and electrostatic interactions between each amino acid of the target protein and the ligand molecule. In addition, we introduced fluctuations in the accessible surface area (ASA) and dihedral angles of the protein-ligand complex system as the entropy terms of the ΔG estimation. The present method included the fluctuation term of structural change of the protein and the effective dielectric constant. We applied this method to 34 protein-ligand complex structures. As a result, the correlation coefficient between the experimental and calculated ΔG values was 0.81, and the average error of ΔG was 1.2 kcal/mol with the use of the fixed parameters. These results were obtained from a 2 nsec molecular dynamics simulation.

## 1. Introduction

The protein-ligand binding free energy (ΔG) has been calculated by various computational methods. Many protein-ligand docking programs have been developed to estimate ΔG [1,2,3,4,5,6,7], but the existing docking software is relatively inaccurate [1,2]. There is an almost 50% success rate of reproducing a protein-ligand complex structure within a root mean square deviation (RMSD) of <2 Å [6,7] and the accuracy of ΔG estimation remains at approximately 2–3 kcal/mol [6,7,8,9,10].

There have been several reports on protein-compound docking and free energy calculation by molecular dynamics (MD) simulation. Even if a protein-ligand complex structure is unknown, *ab initio* MD docking simulations show protein-ligand complex structures and free energy landscapes [11,12,13,14]. Generalized ensemble methods have been adopted for wide conformational searches [15,16,17,18].

In an explicit water model, if a protein-ligand complex structure is known, the binding free energy and the potential of mean force (PMF) along the dissociation path can be obtained by using the filling potential (FP) method [18], the meta-dynamics method [19,20], the smooth reaction path generation (SRPG) method [21], or Jarzynski’s method [22]. We previously proposed the FP and SRPG methods [18,21], each of which generates a reaction path (dissociation path) of the ligand and calculates the free energy surface along the path based on *ab initio* MD simulation. The other trend is the application of Jarzynski’s equation [22]. In this method, a harmonic potential that restrains the ligand at a particular position moves slowly and leads the ligand from the binding state to the dissociation state, and the free energy profile is calculated. Among these methods, MP-CAFFE has been applied to various species, and the ΔG estimation error was almost 1 kcal/mol [23].

The molecular-mechanics Poisson-Boltzmann surface-area (MMPBSA) method [24] and the linear interaction energy (LIE) method [25] have successfully been used to reproduce the trend of ΔGs for a single target protein. These methods are much faster than the *ab initio* MD methods described above. In the LIE method, ΔG is evaluated based on the average van der Waals (vdW) energy and the average electrostatic energy. The weight parameters of the vdW and electrostatic terms are optimized for each target. To apply the LIE method, multiple active compounds and their docking poses are necessary in order to optimize the parameters for each target protein.

The COMBINE method is based on the assumption that biological activities can be correlated with a linear combination of a subset of the van der Waals and electrostatic terms of the interaction energies between a ligand and its surrounding protein residues (such as the target receptor) [26,27]. The protein-ligand binding free energy ΔG is given by:


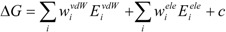
(1)

where E_i_*^vdw^* and E_i_*^ele^* are the van der Waals and electrostatic terms of the interaction energies, respectively, between the ligand and the i-th residue of a protein (the target protein) and *c* is a constant. *w_i_^vdw^* and *w_i_^ele^* are parameters to be determined to reproduce the experimental data.

The coefficients of Equation (1) (*w_i_^vdw^* and *w_i_^ele^*) could be determined by partial least squares (PLS) analysis. As is the case with the LIE, to apply the COMBINE method, multiple active compounds and their docking poses are necessary in order to optimize the parameters for each target protein. It has been shown that the COMBINE analysis predicts binding free energies with good accuracy and also identifies important amino acid residues for the improvement of affinity [26,28,29,30,31,32].

In the present study, we propose a ΔG estimation method based on the direct protein-ligand interaction obtained by molecular dynamics simulation. We introduced the entropy term and the local effective dielectric constant, and modified the van der Waals potential to improve the accuracy of the present method so that it does not require multiple active compounds to predict the ΔG value.

## 2. Results and Discussion

### 2.1. Theoretical Background

ΔG is calculated by Zwanzig equation as follows [33]:


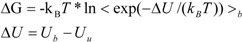
(2)

where U_b_, U_u_, and < >_b_ represent the potential of the protein-ligand bound and unbound states and the average over the bound-state trajectory, respectively. 

Kubo’s cumulant expansion gives the following equation excluding the log and exp functions as [34]:


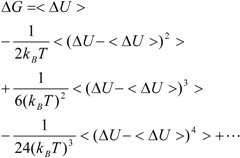
(3)

The first term (linear term) corresponds to the enthalpy, and the higher-order term corresponds to the entropy. The second term becomes:


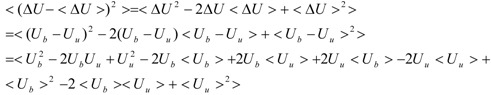
(4)

When we assume:


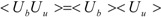
(5)

Then, the second term of Equation (3) becomes: 


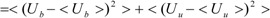
(6)

And Equation (3) becomes:


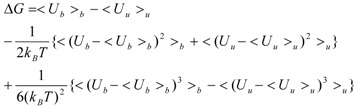
(7)

The linear term (the first and second terms; <U_b_>_b_ − <U_u_>_u_) of Equation (7) corresponds to the LIE approximation, when the energy difference is due to the receptor-ligand and solvent-ligand interaction energies. The LIE approximation calculates ΔG by:


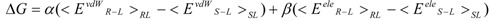
(8)

where E^vdW^_X_ and E^ele^_X_ are the protein-ligand van der Waals interaction energy and the electrostatic interaction energy between the ligand and the surrounding molecules, R-L/S-L represents the interaction of the protein-ligand complex system/solute-ligand system, and the brackets (< >_X_) represent the average over simulation of the protein-ligand complex system (RL) or the solute-ligand system (SL). The LIE equation includes two parameters: α and β. These parameters are known to reproduce the experimental data for each target protein.

The COMBINE approximation calculates the ΔG value by:


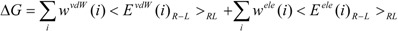
(9)

where the E^vdW^ (i) and E^ele^ (i) are the protein-ligand van der Waals interaction energy and the electrostatic interaction energy between the i-th residue of the protein and the ligand, and w is the parameter. The simulation of the ligand-solution system is not necessary.

Both the LIE approximation without solvent and the COMBINE approximation with the residue-independent w parameter gave the same equation:


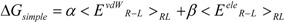
(10)

### 2.2. Entropy Term

In the present study, we introduced the entropy term in Equation (10) as follows. We call this method the direct interaction approximation without solvent (DIAV) method:


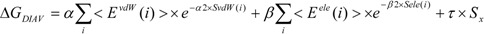
(11)

where E^vdW^(i) and E^ele^(i) are the vdW and electrostatic interactions between the i-th residue of the protein and the ligand, respectively. Svdw(i) and Sele(i) are fluctuations of E^vdW^(i) and E^ele^(i) during the molecular dynamics simulation, respectively. The τ*S_x_ term represents the energy fluctuation of the system corresponding to the second-order term of Equation 7 ((<U_b_ − <U_b_>_b_)^2^).

In Equation (11), the τ*S_x_ term is the fluctuation of energy, but we found that the energy fluctuation itself is not suitable for evaluating ΔG. Instead of the energy, S_x_ is the fluctuation of a property x that is related to the energy. The properties x in the current study are the accessible surface area (x = ASA), the dihedral angles (x = DIH), the vdW potential (x = vdW), and the electrostatic potential (x = ELE) of the protein-ligand complex structure. In the present study, we determine which property is best for estimating ΔG. There are five parameters: α, α2, β, β2, and τ.

### 2.3. Modification of van der Waals Potential Term

To represent the van der Waals (vdW) interaction, a Lennard-Jones (LJ) 12-6-type function is used. In the docking score, the vdW interaction (lipophilic atom contact) term represents both the vdW interaction and the cavity formation energy in solvent; in water, the latter is 10 times greater than the vdW interaction. This function gives very large values when atomic conflicts occur. To reduce these conflicts, an LJ 9-6-type function has been used in a protein-ligand docking study [3]. In general, the absolute value of the vdW interaction is much smaller than the ΔG value. The LJ 12-6 value represents the atomic contact and its hydrophobic interaction. Thus, in the present study, we apply LJ 12-6, LJ 9-6, LJ 8-4, and LJ 6-3-type functions as follows:


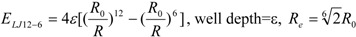
(12)


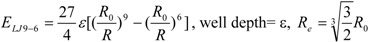
(13)


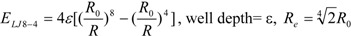
(14)


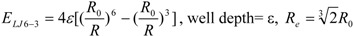
(15)

where R_e_ is the equilibrium distance. The R_e_ and the well depth values are set to the same values obtained from AMBER param99 [35] and the general AMBER force field (GAFF) [36].

The data-sampling MD simulation is performed with the conventional AMBER force field (LJ 12-6 potential), and the analysis is performed using Equations (12)–(15).

### 2.4. Effective Dielectric Constant

In the ligand-binding pocket, the effective dielectric constant (ε_eff_) should be different at each point, since the ε_eff_ values of proteins are 2–4 and the ε_eff_ of water is 78.5. The E^ele^(i) should be scaled by the ε_eff_. We introduced the modification of the electrostatic interaction as follows (we call this method the direct interaction approximation with solvent (DIAS) method):


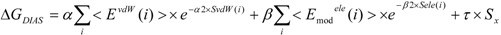
(16)

where E_mod_^ele^(i) is the E^ele^(i) value scaled by the ε_eff_. The ε_eff_ value could be calculated from the ratio between the electrostatic force calculated in the explicit water model and that in vacuum, as follows:


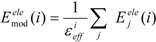
(17)

where E_j_^ele^(i) is the electrostatic interaction between the i-th residue and the j-th atom of the ligand in vacuum. The following scale factor might be a candidate:


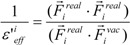
(18)

or:


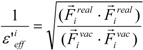
(19)

where F_i_^real^ and F_i_^vac^ are the electrostatic force acting on the i-th atom of the protein considering the solvent and not considering the solvent in the explicit water model, respectively. The F^real^ and F^vac^ were calculated by the molecular dynamics simulation in the explicit water model and in vacuum, respectively.

The scale factor ε’_eff_^i^ by Equation (18) or (19) could be unrealistically large when the denominators of Equations (12) and (13) are nearly zero. Thus, we introduce a parameter *x* and the scale function as follows: 


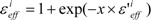
(20)

The value of ε_eff_^i^ in Equation (20) is 1 < ε_eff_^i^, while the actual ε_eff_ value could be less than 1. But we introduced parameter β in Equation (16), thus the actual ε_eff_ parameter is ε_eff_^i^/β. In the following analysis, the factor ε_eff_^i^ in Equation (20) is used as the actual scale factor.

### 2.5. Examination of Entropy Term

We applied the DIAV method (Equation (11)) to the protein-ligand complex structures to examine the entropy property term Sx and performed the leave-one-out cross-validation test, as summarized in Table 1, which also summarizes the optimized parameters. 

**Table 1:** Cross-validation results obtained by Equation (10) and the DIAV method (Equation (11)).

Statistics	ΔG_simple_ (Equation 10)	ELE ^a^	vdW ^a^	ASA ^a^	DIH ^a^
Average error (kcal/mol)	2.22	2.30	1.85	2.06	1.94
R	0.72	0.70	0.72	0.71	0.67
α	0.22	0.22	0.18	0.17	0.15
β	0.017001	0.016411	0.005958	0.012600	0.010430
τ*100	-	−0.078460	−28.506610	−0.026605	−0.000696

The vdW potential is the LJ 12-6 type. a: property (x) of Equation (11). Here α2 = β2 = 0. The energies are presented in kcal/mol, and R represents the correlation coefficient.

In the leave-one-out cross-validation test, one data is selected as the test data that is to be predicted and the other data are used as the teaching data to generate the prediction model equation. The test data is selected one after another in the given data set until all data are selected as the test data. The vdW energy term was set to an LJ 12-6 function, and the dielectric constant was set to 1. The values of the parameters α2 and β2 were set to zero. The ASA parameters (atomic solvation parameter and radius of each atom) were obtained from a previous study [37]. In the present study, the parameters of Equation (11) were optimized by the least-squares deviation error of the ΔG values. Compared to the results obtained by the simplified version of the COMBINE method (Equation (10)), the DIAV method (Equation (11)) slightly improved the accuracy of ΔG. 

### 2.6. Examination of vdW Term

We examined the DIAV method with the vdW term using Equations (12)–(15). The results and the optimized parameters are summarized in Table 2. The ε value was set to 1. The entropy property x was set to the ASA. We also examined the case of x = DIH, and the result was quite similar to that obtained in the case of x = ASA. The LJ 8-4-type function gave the best result among the four functions (LJ 12-6, LJ 9-6, LJ 8-4, and LJ 6-3) in the leave-one-out cross-validation test, while the accuracy obtained was similar among the functions. Thus, the LJ6-3 and LJ4-2−type functions were not used in the following study; instead we focused on the LJ8-4 function.

**Table 2:** Cross-validation results obtained by the DIAV method (Equation (11)) to examine the van der Waals potential type.

Statistics	LJ9-6	LJ8-4	LJ6-3
Average error (kcal/mol)	2.26	1.75	1.89
R	0.69	0.76	0.71
α	0.1727	0.0428	0.0066
β	0.0139	0.0072	0.0078
τ*10000	−2.9273	−2.5677	−2.8531

The energies are presented in kcal/mol, and R represents the correlation coefficient.

The vdW parameters represent both the protein-ligand vdW interaction and the hydrophobic interaction. In the present study, however, the number of data were limited to the optimization of the parameters, and then we used just the original vdW parameters.

### 2.7. Examination of α2 and β2 Parameters

We examined the parameters α2 and β2 of the DIAV method (Equation (11)). The vdW potential was set to the LJ 8-4-type function, and the dielectric constant was set to 1. The entropy property x was set to the ASA. We also examined the case of x = DIH; the result was quite similar to that obtained in the case of x = ASA. The leave-one-out cross-validation results and the optimized parameters are summarized in Table 3. The optimized α2 and β2 were about 0.01 and −0.0013, respectively, and the modulated vdW and electrostatic energy values were close to the original (intact) values. Actually, the parameters α2 and β2 improved the ΔG estimation accuracy, and the equation includes five parameters (α, β, τ, α2, and β2). The two additional parameters (α2 and β2) slightly improved the average accuracy.

### 2.8. Examination of Effective Dielectric Constant Term

We applied the idea of the effective dielectric constant. We applied the DIAS method (Equation (16)) to the estimation of ΔG using the ε_eff_ defined by Equations (18) and (19). The leave-one-out cross-validation results and the optimized parameters are summarized in Table 4.

**Table 3:** Cross-validation results obtained by the DIAV method (Equation (11)) to examine α2 and β2 parameters.

Statistics	ASA	DIH
Average error (kcal/mol)	1.63	1.59
R	0.80	0.76
α	0.04146	0.03832
β	0.00643	0.00491
τ*10000	−2.74887	−0.06949
α2	0.0093	0.0093
β2	−0.0013	−0.0015

The vdW potential is the LJ 8-4 type. The energies are presented in kcal/mol, and R represents the correlation coefficient.

**Table 4:** Cross-validation results obtained by Equation (10), the DIAV (Equation (11)), and the DIAS (Equation (16)) methods.

PDB ID	ΔG_exptl_ (kcal/mol)	ΔG_simple_ (Equation (10)) (kcal/mol)	ΔG_DIAV_ (Equation (11)) (kcal/mol)	ΔG_DIAS_ (Equation (16)) (kcal/mol)
1abe	−9.57	−5.46	−6.27	−6.68
1abf	−7.39	−6.30	−6.67	−6.90
1apu	−10.50	−13.50	−11.98	−11.76
1dbb	−12.27	−8.75	−11.79	−11.69
1dbj	−10.47	−8.35	−12.27	−12.10
1dog	−5.48	−5.40	−6.09	−6.12
1dwb	−3.98	−3.69	−4.83	−5.05
1epo	−10.85	−17.25	−14.82	−15.56
1etr	−10.09	−9.91	−10.35	−10.08
1ets	−11.62	−11.05	−11.82	−11.52
1ett	−8.44	−9.46	−9.99	−9.75
1hpv	−12.57	−14.02	−12.88	−12.78
1hsl	−9.96	−6.53	−6.74	−7.18
1htf	−11.04	−12.45	−11.12	−11.00
1hvr	−12.97	−16.98	−14.67	−14.95
1nsd	−7.23	−7.44	−8.33	−8.13
1pgp	−7.77	−11.01	−11.09	−10.24
1phg	−11.81	−6.88	−8.03	−8.22
1ppc	−8.80	−9.83	−8.66	−8.85
1pph	−8.49	−8.50	−7.87	−8.00
1rbp	−9.17	−9.29	−8.58	−8.91
1tng	−4.00	−4.15	−4.64	−4.90
1tnh	−4.59	−3.54	−4.24	−4.61
1ulb	−7.23	−3.82	−5.71	−5.74
2cgr	−9.92	−7.07	−10.94	−10.88
2gbp	−10.36	−8.95	−9.27	−9.77
2ifb	−7.41	−9.57	−8.53	−8.38
2phh	−6.38	−4.09	−6.83	−6.79
2r04	−8.48	−10.39	−10.31	−10.26
2tsc	−11.62	−11.05	−8.68	−8.28
2ypi	−6.58	−5.40	−5.72	−6.45
3ptb	−6.46	−4.93	−5.02	−4.55
4dfr	−13.23	−11.52	−13.93	−13.52
5abp	−9.05	−6.64	−7.19	−7.59
Averageerror	-	1.88	1.30	1.22
R	-	0.73	0.81	0.81
α	-	0.0503	0.0378	0.0307
β	-	0.0125	0.0082	0.0118
τ∗10000	-	-	−2.4178	−2.4312
α2	-	-	0.0093	0.01
β2	-	-	−0.0011	−0.00312
x	-	-	-	0.6

The vdW potential is the LJ 8-4 type. The property x of Sx is the ASA. The energies are presented in kcal/mol, and R represents the correlation coefficient.

**Figure 1:**
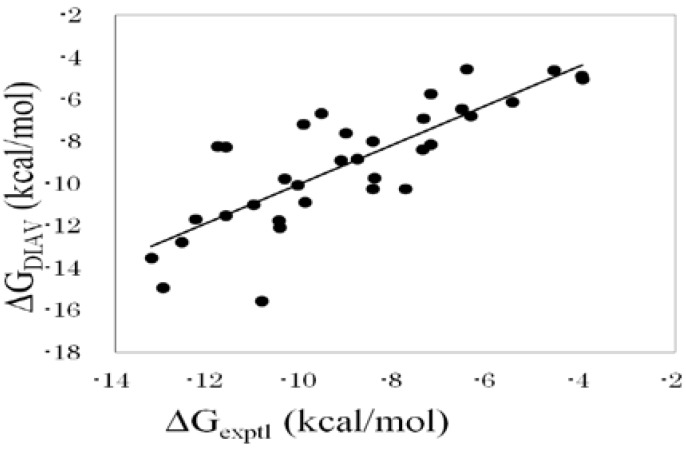
Cross-validation results obtained by the DIAV method. The experimental data (ΔG_exptl_) and the calculated value (ΔG_DIAV_).

As with the results described above, the best property x among the four properties (ASA, DIH, vdW, and ELE) was the ASA. The DIAS results obtained by Equation (19) were better than those obtained by Equation (18). The DIAS results in Table 4 were obtained by using Equation (19). The consideration of ε_eff_ slightly improved the ΔG estimation. As a result, the correlation coefficient between the experimental and the calculated ΔG values was 0.81, and the average error of ΔG was 1.2 kcal/mol. This result greatly improved the results obtained by Equation (10). Figure 1 shows the correlation between experimental and calculated ΔG values obtained by the DIAS method (Equation (16)).

We examined the time-dependency of the ΔG obtained by the DIAS method. After 1 nsec MD simulation for equilibration, the sampling runs of 0.5 nsec, 1 nsec, 1.5 nsec and 2 nsec were performed. The ΔG values did not depend on the sampling-time length so much. Namely, the average error over the 34 target protein-ligand complexes were 1.43 kcal/mol, 1.22 kcal/mol, 1.23kcal/mol and 1.23 kcal/mol for the 0.5 nsec, 1 nsec, 1.5 nsec and 2nsec sampling times, respectively. The initial structures of these simulations were the experimentally obtained protein-compound complex structures. Thus, the protein-compound interaction did not depend on the sampling-time length so much.

The results showed that the current method worked well for various target proteins. This method could be extended as:


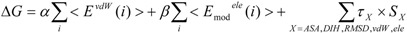
(21)

where α, α_2_, β, β_2_, and τ_x_ are the parameters. This extension is one of the generalized forms of Equation (16). We examined the combination of two properties out of five. The averaged error was increased by the combination of two entropy terms. Thus, Equation (16) is simple and accurate compared to Equation (21).

We applied the generalized Born surface area (GBSA) method [37,38,39] for the ΔG calculation to the same protein-compound set used in the current study. The average error and the correlation coefficient between the experimental and calculated ΔG values were 51.7 kcal/mol and 0.03 that showed very weak correlation, respectively. The GBSA method is good to reproduce the trend of ΔG values of many ligands for one target protein. In the current study, each target protein has only one or a few ligands. The error of the ΔG obtained by the GBSA method is large, thus, the GBSA method could not reproduce the trend of the ΔG value in the current study. In this examination, the DIAS/DIAV methods showed the better results than the GBSA method.

### 2.9. Application to Docking-Pose Prediction

To evaluate the present method, we applied the DIAS method to the protein-ligand docking pose prediction. Usually, only 20%–30% of the docking poses generated by the protein-ligand docking program are correct (RMSD < 2 Å) in the cross-docking test, whereas 50%–70% of the docking poses generated by the protein-ligand docking program are correct (RMSD < 2 Å) in the self-docking test [7]. Of course, the cross-docking test is necessary for practical evaluation of the protein-ligand docking. In this section we mimicked the cross-docking test. We selected the docking poses by both the DIAS method (Equation (16)) and the docking program (Sievgene/myPresto [7]), then compared the results. The docking score of Sievgene was determined as:


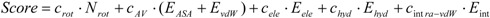
(22)

where *N_rot_, E_ASA_, E_vdW_, E_ele_, E_hyd_*, and *E_intra-vdW_* represent the number of rotatable bonds of the ligand molecule, the hydrophobic energy due to the accessible surface area, the vdW energy, the protein-ligand Coulombic potential, the hydrogen bond energy, and the intramolecular vdW energy of the ligand for Sievgene [7]. Also, *c_rot_, c_AV_, c_ele_, c_hyd_*, and *c_intra-vdW_* are the optimized coefficient for each energy term. For each atom type, the sum of *E_ASA_* and *E_vdW_* gives a grid potential, and both energy terms are always simultaneously calculated. Thus, these two terms share the same coefficient, *c_AV_*. Sievgene utilizes the grid potential to calculate each energy term except for the intramolecular interactions. In this study, a mesh size of 60 × 60 × 60 was adopted.

In this test, we prepared three types of protein structures: (model 1) the intact protein structure prepared in Section 2, (model 2) the energy-minimized structure of apo protein in water, and (model 3) the final structure of 2-nsec MD simulation of apo protein in water. The Sievgene docking program generated five docking poses for each target protein of the three prepared structures (models 1–3). Then each protein-ligand complex structure was evaluated by the DIAS (Equation (16)) with the fixed parameter described in Table 5 in the same manner described in the previous section (the vdW function was the LJ 8-4 type function, and the property x of Sx was the ASA). The best score poses were selected by Sievgene based on docking score, and the best ΔG poses were selected by DIAS. The results are summarized in Table 5.

**Table 5:** Docking accuracy.

Initial structure (intact PDB coordinates: model 1)	Top ΔG structure by the DIAS method	Top scoring structure by Sievgene	Best among the top 5 structures
RMSD < 1 Å	29.4%	35.3%	47.1%
RMSD < 2 Å	41.2%	76.5%	94.1%
RMSD < 3 Å	47.1%	94.1%	94.1%
**Energy-minimized structure** ** (model 2)**	**Top ** **ΔG structure by the DIAS method**	**Top scoring structure by Sievgene**	**Best among the top 5 structures**
RMSD < 1 Å	40.0%	6.7%	66.7%
RMSD < 2 Å	73.3%	46.7%	93.3%
RMSD < 3 Å	80.0%	73.3%	93.3%
**Structure after MD simulation** ** (model 3)**	**Top ** **ΔG structure by the DIAS method**	**Top scoring structure by Sievgene**	**Best among the top 5 structures**
RMSD < 1 Å	20.0%	0.0%	0.0%
RMSD < 2 Å	33.3%	33.3%	33.3%
RMSD < 3 Å	53.3%	46.7%	66.7%

The vdW potential is the LJ 8-4 type. The property x of Sx is the ASA.

When the energy-minimized structures (model 2) were used, the results obtained by the DIAS method were much better than the Sievgene results. The DIAS method selected the correct poses at a rate of 73% (RMSD < 2 Å). Even if the DIAS method selected the best docking poses among the five poses generated by Sievgene, 93% of the five generated poses satisfy the RMSD < 2 Å. Thus, the DIAS method selected 78% (73% out of 93%) of the correct poses. This shows that the DIAS method is useful for practical pose prediction in drug design.

When the initial structures (model 1) were used, the Sievgene results were better than the results obtained by the DIAS method. This is a trivial self-docking test, and the MD simulations for energy calculation should slightly change the ligand coordinates from the crystal structures by thermal fluctuation. When the final structures of the MD simulation (model 3) were used, only 33.3% of the docking poses were correct (RMSD < 2 Å) by Sievgene and the DIAS method. Still, the results obtained by the DIAS method were better than those obtained by Sievgene. The shapes of the ligand-binding pockets should be changed from their suitable structures after the MD simulations. This model structure is not suitable for docking studies.

## 3. Data Preparation

To determine the coefficients for the ΔG score, we performed a protein-ligand docking simulation based on the known complex structures registered in the Protein Data Bank. Here, 34 complexes accompanied by the experimental binding free-energy values were selected from the database that was used to determine the ΔG scores of the PRO_LEADS [6]. The PDB identifiers and the names are summarized in Table 6. In the test dataset, the metalloproteins were removed from the present analysis. Metal atoms (Zn and Fe atoms) formed covalent bonds with O and S atoms of the ligands, and the classical force field that we applied could not represent the covalent bond. Thus, the present method cannot calculate ΔG values for metalloproteins with high precision.

**Table 6:** List of the proteins used.

PDB ID	Protein
1abe	L-ARABINOSE-BINDING PROTEIN
1abf	L-ARABINOSE-BINDING PROTEIN
1apu	ACID PROTEINASE (PENICILLOPEPSIN)
1dbb	FAB' FRAGMENT
1dbj	FAB' FRAGMENT
1dog	GLUCOAMYLASE
1dwb	THROMBIN
1epo	ENDOTHIA ASPARTIC PROTEINASE
1etr	THROMBIN
1ets	THROMBIN
1ett	THROMBIN
1hpv	HIV-1 PROTEASE
1hsl	HISTIDINE-BINDING PROTEIN
1htf	HIV-1 PROTEASE
1hvr	HIV-1 PROTEASE
1nsd	NEURAMINIDASE
1pgp	6-PHOSPHOGLUCONATE DEHYDROGENASE
1phg	CYTOCHROME P450
1ppc	TRYPSIN
1pph	TRYPSIN
1rbp	RETINOL-BINDING PROTEIN
1tng	TRYPSIN
1tnh	TRYPSIN
1ulb	PURINE NUCLEOSIDE PHOSPHORYLASE
2cgr	IGG2B (KAPPA) FAB FRAGMENT
2gbp	D-GALACTOSE/D-GLUCOSE-BINDING PROTEIN
2ifb	INTESTINAL FATTY ACID BINDING
2phh	P-HYDROXYBENZOATE HYDROXYLASE
2r04	RHINOVIRUS 14 (HRV14)
2tsc	THYMIDYLATE SYNTHASE
2ypi	TRIOSE PHOSPHATE ISOMERASE
3ptb	TRYPSIN
4dfr	DIHYDROFOLATE REDUCTASE
5abp	L-ARABINOSE-BINDING PROTEIN

The structural ensembles generated from the PDB structure given by MD in explicit water were prepared as follows. All target proteins were prepared with ligands (protein-ligand complex structure). The force fields and the charges of the protein atoms originated from AMBER parm99 [35]. The atomic charge of each ligand was determined by the restricted electrostatic point charge (RESP) procedure using HF/6-31G*-level quantum chemical calculations [40]. We used Gaussian98 to perform the quantum chemical calculations [41]. The whole structure of each protein was embedded in a sphere of TIP3P [42] water (CAP water), including ion particles of 0.1% Na^+^ and Cl^−^, in order to neutralize the total charge of the systems. The center of the sphere was set at the mass center of the protein. The shortest distance between the protein atom and the CAP sphere wall was set to 10 Å. Before an MD calculation was performed for the entire system, an MD calculation for only the solvent parts (solvent water and counter ions) was performed with the protein, ligand, and metal ion coordinates fixed, so as to bring the solvent parts sufficiently close to an equilibrium state. The SHAKE method was used to constrain covalent bonds between heavy and hydrogen atoms in any molecule in the system [43]. MD simulations of the entire system were performed using 2.0 fsec time steps with the temperature set at 310 K; the fast multipole method [44] was used to calculate the Coulombic interaction. The cutoff distance of the van der Waals interaction was 12.0 Å. The MD simulations were performed by using cosgene/myPresto [18]. After equilibration steps of 1,000 psec, the protein coordinates were sampled every 1 psec. Finally, we obtained 1,000 structures for each target protein in the 1,000 psec production run. The software program myPresto version 4 [45] was used for the simulation.

## 4. Conclusions

We have developed the direct interaction approximation (DIA) method and examined both the direct interaction approximation without solvent (DIAV) and with solvent (DIAS) methods. The results showed that the inclusion of the fluctuation of the ASA/dihedral angle terms drastically improved the accuracy of ΔG. The DIAV method (Equation (16)) was the final form for the simple and accurate estimation of ΔG. The effective dielectric constant should be calculated by Equations (19) and (20), and the vdW potential should be the LJ 8-4-type function. This equation included six parameters: α, β, α2, β2, τ, and x. The six optimized parameters could be applied to all of the target proteins.

In the explicit water model, the DIA (DIAV and DIAS) methods required only the MD simulation of the protein-ligand complex. The DIA method with the LJ 8-4-type function improved the accuracy of the calculated ΔG value drastically: the correlation coefficient between the experimental and the calculated ΔG values was improved to 0.8 as obtained by the DIAV method, from 0.7 as obtained by the simplified COMBINE method without the entropy term (Equation (10)), and the average error of ΔG was improved to 1.2 kcal/mol as obtained by the DIAS method, from 1.9 kcal/mol as obtained by Equation (10).
